# Global Molecular Epidemiology of *Cryptococcus neoformans* and *Cryptococcus gattii*: An Atlas of the Molecular Types

**DOI:** 10.1155/2013/675213

**Published:** 2013-01-09

**Authors:** Massimo Cogliati

**Affiliations:** Lab. Micologia Medica, Dipartimento di Scienze Biomediche per la Salute, Università degli Studi di Milano, Via Pascal 36, 20133 Milano, Italy

## Abstract

Cryptococcosis is a fungal disease affecting more than one million people per year worldwide. The main etiological agents of cryptococcosis are the two sibling species *Cryptococcus neoformans* and *Cryptococcus gattii* that present numerous differences in geographical distribution, ecological niches, epidemiology, pathobiology, clinical presentation and molecular characters. Genotyping of the two *Cryptococcus* species at subspecies level supplies relevant information to understand how this fungus has spread worldwide, the nature of its population structure, and how it evolved to be a deadly pathogen. At present, nine major molecular types have been recognized: VNI, VNII, VNB, VNIII, and VNIV among *C. neoformans* isolates, and VGI, VGII, VGIII, and VGIV among *C. gattii* isolates. In this paper all the information available in the literature concerning the isolation of the two *Cryptococcus* species has been collected and analyzed on the basis of their geographical origin, source of isolation, level of identification, species, and molecular type. A detailed analysis of the geographical distribution of the major molecular types in each continent has been described and represented on thematic maps. This study represents a useful tool to start new epidemiological surveys on the basis of the present knowledge.

## 1. Introduction

According to the last report (December 2010) from the Joint United Nations Program on HIV/AIDS and the World Health Organization (http://www.unaids.org/), 34 million people worldwide suffer from HIV infection/AIDS, 2.7 million people are newly infected every year from this disease, and 1.8 million people die from AIDS-related causes. Neuropathological conditions are present in approximately 70% to 90% of AIDS patients [1]. Cryptococcal meningitis is considered an AIDS-defining condition [2–4], and it is the most common fungal infection of the central nervous system and the third most frequent neurological complication in AIDS patients [1]. The main etiological agents of cryptococcosis are the basidiomycetous yeasts *Cryptococcus neoformans* and *Cryptococcus gattii* which can also infect, although with a significantly lower incidence, people with decreased immunity such as individuals with sarcoidosis, lymphoproliferative disorders, those undergoing immunosuppressive therapies [4–6], and more rarely immunocompetent people [7, 8]. Worldwide, *C. neoformans* and *C. gattii* infections cause an estimated one million cases of cryptococcal meningitis per year among people with HIV/AIDS, resulting in nearly 625,000 deaths (Centers for Disease Control and Prevention, CDC, Atlanta, USA, http://www.cdc.gov/). The greatest burden of disease occurs in sub-Saharan Africa, where mortality is estimated to be 50% to 70% [9, 10]. In the United States and other developed countries, cryptococcosis is decreasing among persons with HIV/AIDS due to the availability of high active antiretroviral therapy, and, at present, the mortality is around 12% (CDC, Atlanta, USA).

Although in the past the etiological agent of cryptococcosis was considered a homogeneous anamorphic species (*C. neoformans*), now the two species *C. neoformans* and *C. gattii* have been separated on the basis of numerous differences such as geographical distribution, ecological niches, epidemiology, pathobiology, clinical presentation, and molecular characters. *C. neoformans* species is further classified in two varieties, *C. neoformans* var. *grubii* (serotype A) and *C. neoformans* var. *neoformans* (serotype D), which are also able to recombine and to produce diploid or aneuploid intervarietal AD hybrids [78, 131, 132]. *C. neoformans* has been widely associated to avian excreta [44, 97, 98, 133–136] although it has been isolated also from other sources [11, 28, 60, 99, 100, 137].

*C. gattii* species is classified in two different serotypes, B and C, which have not yet elevated to the variety status. This species was thought to be restricted to tropical and subtropical regions but a recent outbreak due to *C. gattii* infection, which occurred in Vancouver Island and North West Pacific Coast of America [121, 138], has expanded the geographical area of this pathogen also to temperate regions. In addition, interspecies hybrids between *C. neoformans* and *C. gattii* have been found in Colombia, Brazil, India, and The Netherlands [29, 122, 139, 140]. 

Besides a prevalent asexual life cycle, both species have also a bipolar sexual cycle with two mating types, MATa and MAT*α*, the latter being the most prevalently isolated from both patients and environment. *Filobasidiella neoformans* and *Filobasidiella bacillispora* are the sexual states of *C. neoformans* and *C. gattii*, respectively [141, 142]. During sexual recombination, either filaments with clamp connections and basidiospores are produced [143]. Recombinant basidiospores are also produced via same-sex mating [144] and are thought to be the propagules responsible for the infection of the host [143].

The availability of whole genome sequences from *C. neoformans* and *C. gattii* strains and the recent progresses in the molecular biology have greatly advanced our understanding of this pathogenic yeast [145, 146]. The disease aspects of cryptococcal infection are becoming better defined, while the life cycle of this fungus in the environment remains less well established. How this fungus has spread worldwide, the nature of its population structure, and how it evolved to be a deadly pathogen are ongoing research subjects that are key to our understanding of this environmental pathogen. 

Due to the importance of the *C. neoformans*/*C. gattii* species complex as human fungal pathogens, several research groups are currently focusing on the molecular determination of the number of genetically diverged subgroups within each species. Several molecular typing techniques have been applied: multilocus enzyme electrophoresis (MLEE) [147]; DNA fingerprinting [148]; random amplification of polymorphic DNA (RAPD) [12, 149]; PCR fingerprinting [150]; amplified fragment length polymorphism (AFLP) [11]; restriction fragment length polymorphism (RFLP) of *PLB1* [13], *GEF1* [37], or *URA5* genes [64]; sequencing of ITS1-5.8S-ITS2 rDNA region [38] or intergenic spacer region (IGS) [151] and, more recently, multilocus sequence typing (MLST) [14, 45, 53]; multilocus microsatellite typing (MLMT) [89, 140]; matrix-assisted laser desorption ionization-time of flight mass spectrometry-based method (MALDI-TOF) analysis [79, 152, 153].

The multitude of data obtained with different typing methods has raised the problem to compare the results and the need to standardize genotypes nomenclature among comparable methods. The first steps towards this direction have been recently achieved by the comparison of the results obtained using the most common *Cryptococcus* typing methods [15] and by the identification of eight major molecular types among the two *Cryptococcus* species. A second step was to standardize a gold standard typing method able to produce unambiguous and comparable results [154]. Finally, a global database was implemented in order to collect the different genotypic profile and to make the data available for the research community [154]. This task is the main aim of the activity of the ISHAM *Cryptococcus* working Group for “Genotyping of *Cryptococcus neoformans* and *Cryptococcus gattii*” which promotes the genotyping of the two *Cryptococcus* species to elucidate the global epidemiology of this life-threatening pathogen.

## 2. Standardization of *Cryptococcus* Molecular Typing Methods

Although numerous molecular techniques have been applied to subtype *C. neoformans* and *C. gattii* strains, only three methods were proved to produce comparable results: PCR fingerprinting, AFLP, and MLST. 

PCR fingerprinting is based on the amplification of DNA sequences flanked by simple DNA repeats which are used as single primers in the PCR. The amplification produces a banding profile that discriminates the strains at subspecies level. The primers employed in PCR fingerprinting include the minisatellite-specific core sequence of wild-type phage M13 (5′-GAGGGTGGCGGTTCT-3′) and the microsatellite-specific primer (GACA)_4_. The technique was first applied to *Cryptococcus* typing in 1993 [150] to study a set of cryptococcal strains. A high polymorphism was detected among the investigated strains which could be separated in two groups corresponding to serotype A and serotype D and a third one including both serotypes B and C. In a different study, a variety of genotyping clusters was identified during the investigation of some Italian *C. neoformans* isolates by (GACA)_4_ PCR fingerprinting [155]. The results showed a strong correlation between genotypes and serotypes. The more prevalent genotype was named VN1 and corresponded to *C. neoformans* var. *neoformans*, serotype D, a second genotype was identified as VN6 and corresponding to *C. neoformans* var. *grubii*, serotype A, and further two genotypes (VN3 and VN4) included isolates with a banding pattern intermediate between VN1 and VN6 suggesting that these strains were AD hybrids. Therefore, this study provided, for the first time, a tool to identify unambiguously intervarietal AD hybrids. In a first attempt to standardize the technique, PCR fingerprinting, using either M13 (GACA)_4_ primer, and RAPD were applied to genotype 356 *C. neoformans* global isolates [15]. Both typing methods were able to identify four different molecular types: VNI and VNII corresponding to *C. neoformans* var. *grubii*, serotype A, VNIV corresponded to *C. neoformans* var. *neoformans*, serotype D, and VNIII including all AD hybrids. Later, a collaborative network between Spanish and Latin American researchers was established [64], and the 340 isolates collected were investigated by M13 PCR fingerprinting and *URA5* RFLP. The results showed, for the first time, the distribution of eight molecular types in the studied countries. The molecular types VNI, VNII, VNIII and VNIV were recognized among the *C. neoformans* isolates, as previously reported, and further four molecular types, VGI, VGII, VGIII, and VGIV, were found among *C. gattii* isolates. PCR fingerprinting was then applied in several studies, and, at present, thousands of strains from different countries of the world have been characterized using this typing method [14, 30, 37, 39, 56, 78, 80, 93, 97, 101, 120, 156–164].

The AFLP typing method is based on digestion of DNA samples with a frequent and a rare cutting endonuclease enzyme combined with amplification using an adaptor that creates specificity at the restriction sites. Subsequent rounds of PCR are able to select a unique profile depending on the number of nucleotides added to the primers. Fluorescently labeled fragments are separated by an automated capillary sequencer and visualized as a virtual banding profile [165]. Application of this technique to *Cryptococcus* typing requires a digestion with *Mse*I and *Eco*RI restriction enzymes and an amplification with the two selective primers *Mse*I-G and *Eco*RI-AC [11]. The analysis of 207 global *C. neoformans* and *C. gattii* isolates led to identify three AFLP genotypes (AFLP1, APLP2, and AFLP 3) among *C. neoformans* strains and three (AFLP4, AFLP5, and AFLP6) among *C. gattii*. In addition, two further subtypes of the genotype AFLP1 were identified as AFLP1A and AFLB1B [11]. Since AFLP is a technique with a high discriminatory power, being able to assign a unique profile to each strain, it contributed to elucidate the cause of the outbreak of *C. gattii* in Vancouver Island [157]. In the study, two AFLP6 subtypes were clearly identified as the cause of the outbreak: AFLP6A and AFLP6B. Since AFLP6A was isolated in 75% of the cases in Vancouver Island environment and AFLP6B isolates were less frequent, it was hypothesized that the former genotype was more virulent than the latter. The higher virulence of AFLP6A than AFLP6B was actually shown later in murine model [14]. Finally, AFLP application to *Cryptococcus* typing contributed to discover new interspecies hybrids between *C. neoformans* and *C. gattii.* Both AFLP1/AFLP4 (AFLP9) and AFLP3/AFLP4 (AFLP8) hybrid isolates were identified during some studies carried out in The Netherlands [122, 139, 140].

MLST is a typing technique based on sequence analysis of a set of polymorphic loci. The combination of the different allele types of the selected loci determines the MLST genotype [166]. One hundred and two global *C. neoformans* var. *grubii* isolates were analyzed by MLST in a first study which employed the following set of 12 loci: *CAP10*, *CAP59*, *GPD1*, *LAC1*, *MPD1*, *MP88*, *SOD1*, *TEF1*α**, *TOP1*, *URE1,* and IGS1 [53]. The results showed two major clades among the studied isolates, corresponding to PCR fingerprinting molecular types VNI and VNII and a third new clade, VNB, including only isolates from Botswana.

A second MLST study investigated 202 global *C. gattii* isolates in order to elucidate the origin of Vancouver Island outbreak isolates [14]. MLST analysis, using seven loci (*CAP10*, *GPD1*, IGS1, *LAC1*, *MPD1*, *PLB1*, and *TEF1*α**) and the two mating-type specific loci *SXI*α** and *SXIa*, was able to differentiate all the four PCR fingerprinting molecular types, VGI-VGIV, as well as both Vancouver Island outbreak subtypes, VGIIa and VGIIb.

Although others studies have been carried out using alternative MLST schemes [31, 73, 167], the research community involved in *C. neoformans* and *C. gattii* genotyping has reached an agreement to adopt a common MLST scheme based on the two main studies reported earlier. During a meeting in 2007, the ISHAM *Cryptococcus* working group members established to adopt MLST as the gold standard technique for *C. neoformans* and *C. gattii* molecular typing [154]. The standard MLST scheme includes the sequencing of seven loci, *GPD1*, IGS1, *CAP59*, *LAC1*, *SOD1*, *PLB1*, and *URA5*, which combined represent the minimum number of genes giving the maximum discrimination power. In addition, genotype nomenclature between the three main typing methods (PCR fingerprinting, AFLP, and MLST) was compared and standardized as reported in Table 1, where reference strains are also indicated.

The standard MLST scheme was applied in some recent studies contributing to identify new MLST genotypes. The investigation of 13 Korean *C. neoformans* var*. grubii* isolates led to the identification of a clonal population, designated genotype VNIc, which was prevalently isolated from non-AIDS patients [99]. The same finding seems to be confirmed by other authors who analyzed 35 isolates from Japanese non-HIV patients with cryptococcosis [100]. More recently, a large MLST study [121], carried out on 183 *C. neoformans* var. *grubii* Thai clinical isolates, revealed a low diversity of this population compared to that found in Africa and the Americas. The analysis showed also that the MLST data were consistent with a proposed ancestral African origin of *C*. *neoformans* var. *grubii.* MLST profiles of 107 Ugandan *C. neoformans* var. *grubii* clinical isolates were shown to be associated with the host immunological response providing a new tool to predict virulence [57]. Finally, four serotype-C VGIV *C. gattii* clinical isolates were identified in India, and their MLST profiles were found to be strictly correlated to those from South African VGIV isolates [34]. In order to include all *C. neoformans* var. *grubii* sequences and genotypes obtained using the standard MLST scheme, a preliminary MLST database was constructed at the Imperial College of London (London, UK, http://www.mlst.net/). Unfortunately, this database has the limit to require a long time for sequence check before the sequences could be included and assigned with the right sequence type code. To overcome these limits, a new MLST database has been established at the Molecular Mycology Research Laboratory (University of Sydney, Sydney, Australia, http://www.mycologylab.org/). The system assigns the sequence code automatically when a sequence is compared with the database and a progressive sequence code to new sequences. Subsequently, the users are required to send all the data necessary for quality control as well as the clinical data to complete the database. At present, the *C. neoformans* var. *grubii* database contains 355 strains with 110 sequence types, and the *C. gattii* database contains 400 strains with 160 sequence types [169].

## 3. Combined Epidemiological Analysis

A total of 68,811 *C. neoformans* and *C. gattii* isolates, reported by hundreds of global research studies, were analyzed. Data search was performed in PubMed database (http://www.ncbi.nih.gov/) using the keyword “cryptococcus” combined with a country name, that is, “cryptococcus italy.” Each reference from the resulting list of references was selected if it reported data concerning the isolation of one or more *Cryptococcus* species complex isolates. Isolates reported without an identification code or without a citation were considered new isolates and included in the analysis, whereas isolates reported from more than one paper were considered only once. Then, all the isolates were analyzed on the basis of their geographical origin, source of isolation, level of identification, species, and molecular type.

### 3.1. Oceania

**Figure 1:**
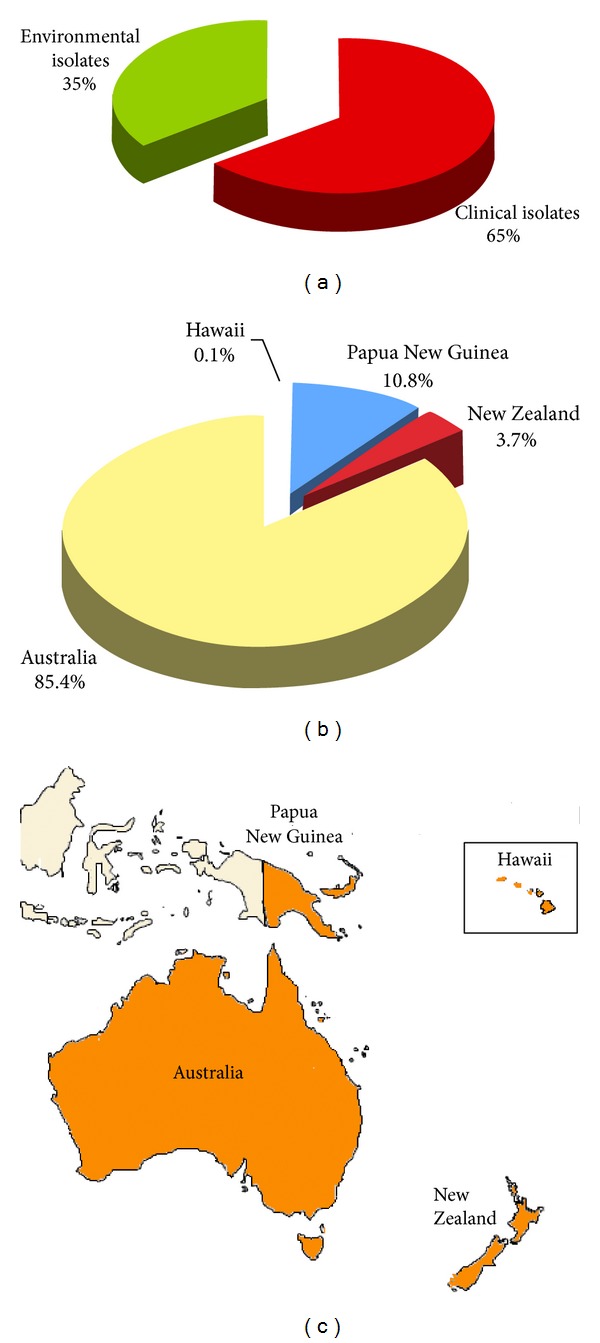
Percentage of *Cryptococcus neoformans* and *Cryptococcus gattii* isolates from clinical (*n* = 1,594) and environmental (*n* = 924) sources (a). Distribution of *Cryptococcus neoformans* and *Cryptococcus gattii* isolates in the different countries of Oceania (b). Map of the geographical distribution of the *Cryptococcus neoformans* and *Cryptococcus gattii* isolates in Oceania (c). Clinical isolates were reported from red-colored countries, whereas both clinical and environmental isolates were reported from orange-colored countries.

A total of 2,518 *Cryptococcus* species complex isolates were reported from four countries of Oceania: Australia, New Zealand, Papua New Guinea, and Hawaii Islands. Most of the strains were isolated in Australia representing 85.4% of the isolates reported. Sixty-five percent of the isolates were from clinical source, whereas 35% were from environmental and veterinary sources (Figure 1). *C. neoformans* was isolated from cat, dog, horse, koala, ferret, *Potorous gilbertii* [16, 170–173], and from *Eucalyptus camaldulensis* and pine needles [11], while *C. gattii* was isolated from kiwi, cat, dog, horse, sheep, cow, koala, quokka, cockatoo, ferret, *Potorous tridactylus*, echidna, African grey parrot, and dolphin [17–19, 174, 175], and from *Eucalyptus camaldulensis*, *Eucalyptus tereticornis*, *Syncarpia glomulifera*, insect frass, olive seedlings, and plant debris [11, 20, 21, 176].

**Figure 2:**
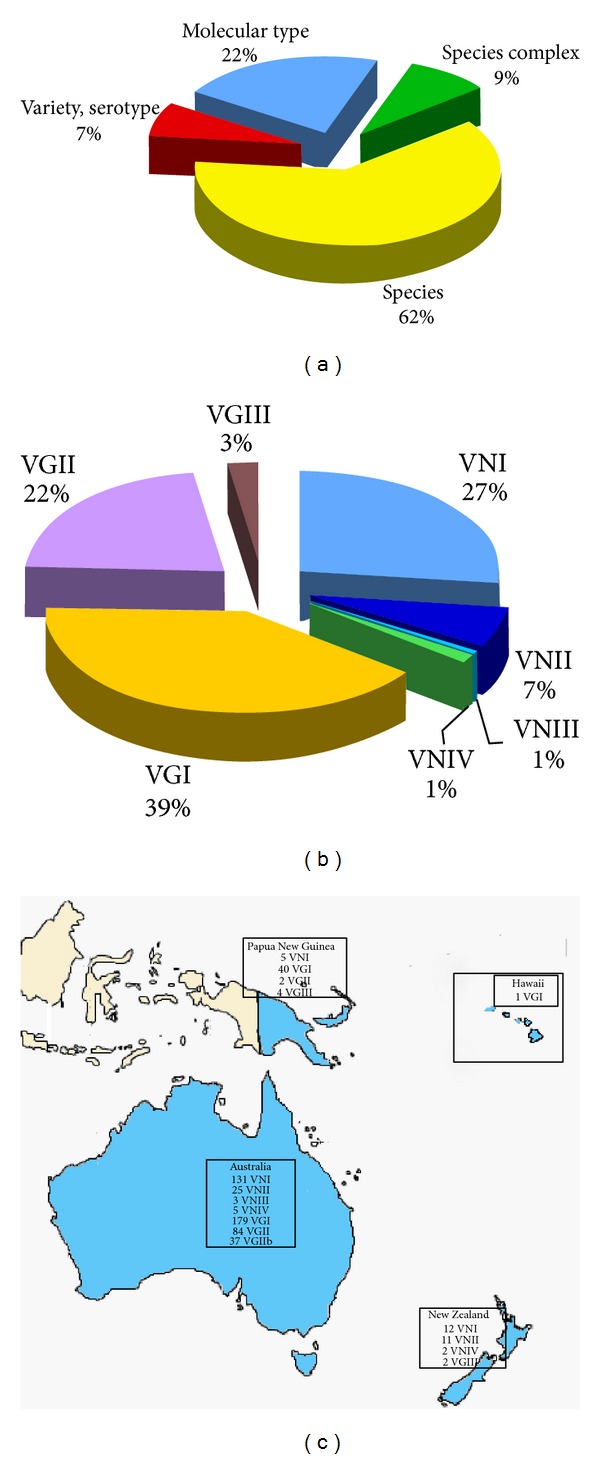
Percentage of *Cryptococcus neoformans* and *Cryptococcus gattii* isolates (*n* = 2,518) identified at species complex, species, variety/serotype, or molecular type level (a). Prevalence of the different VN and VG molecular types among the isolates identified at molecular type level (*n* = 543) (b). Geographic distribution of the molecular types identified in Oceania (c). Molecular typing data have been combined from the following references: Australia [11–27], New Zealand [13, 15], and Papua New Guinea [13–15, 23, 25].

Identification at species level was performed for 62% of the isolates, variety or serotype was identified in 7%, and molecular type in 22% (*n* = 543). Only a small percentage of the isolates (9%) was identified as *Cryptococcus* species complex (Figure 2). A total of 1,328 *C. gattii* and 900 *C. neoformans* isolates were reported. Among the isolates identified at molecular type level, VGI represented the more frequently isolated molecular type (39%) followed by VNI (27%) and VGII (22%), the other molecular types were less frequent. No VGIV isolates have been yet isolated from Oceania.

Figure 2 shows the molecular type geographical distribution in the different countries of Oceania. Although *C. gattii*, with molecular types VGI, VGII, and VGIII, is prevalent in Australia and in Papua New Guinea, only two VGIII isolates were reported from New Zealand where, on the contrary, *C. neoformans* (VNI, VNII, and VNIV) is prevalently isolated.

### 3.2. Asia

**Figure 3:**
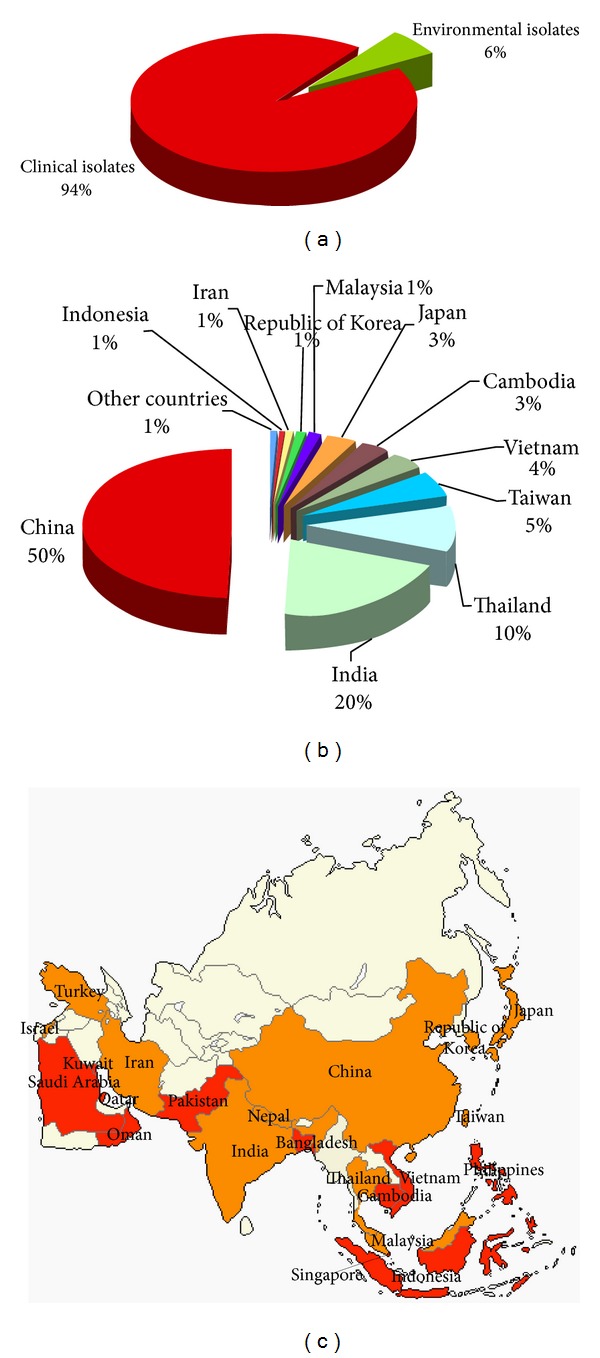
Percentage of *Cryptococcus neoformans* and *Cryptococcus gattii* isolates from clinical (*n* = 18,412) and environmental (*n* = 1,239) sources (a). Distribution of *Cryptococcus neoformans* and *Cryptococcus gattii* isolates in the different Asian countries (b). Map of the geographical distribution of the *Cryptococcus neoformans* and *Cryptococcus gattii* isolates in Asia (c). Clinical isolates were reported from red-colored countries, whereas both clinical and environmental isolates were reported from orange-colored countries.

The combined analysis including all the Asian countries showed that a total of 19,651 *C. neoformans* and *C. gattii* isolates were reported. China, India, and Thailand, together, mainly contributed to the study reporting the 80% of the Asian isolates. Six percent of the isolates were recovered from the environment or from animals in Turkey, Israel, Iran, India, Nepal, China, Thailand, Malaysia, Taiwan, Republic of Korea, and Japan (Figure 3). In most of the environmental surveys, *C. gattii* was isolated from tree samples, namely, from *Syzygium cumini*, *Mimusops elengi*, *Azadirachta indica*, *Acacia nilotica*, *Cassia fistola*, *Manikara hexandra*, *Polyalthia longifolia*, *Eucalyptus camaldulensis*, *Tamarindus indica*, *Cassia marginata*, and *Mangifera indica* [32, 33, 177], while the only ten isolates from an animal source were recovered from koalas living in two different zoos in Japan [178, 179]. On the contrary, *C. neoformans* was prevalently isolated from pigeon and other birds excreta [180] and less frequently from trees such as *Eucalyptus* tree, *Tamarindus arjuna*, *Tamarindus indica*, *Cassia fistola*, *Syzygium cumini*, and *Ficus religiosa* [33, 177, 181, 182], as well as from some vegetables and fruit (tomato, carrot, banana, eggplant, papaya, apple, and guava) [183, 184]. Among animals, few *C. neoformans* isolates were isolated from cat and dog and one from a bandicoot [185, 186].

**Figure 4:**
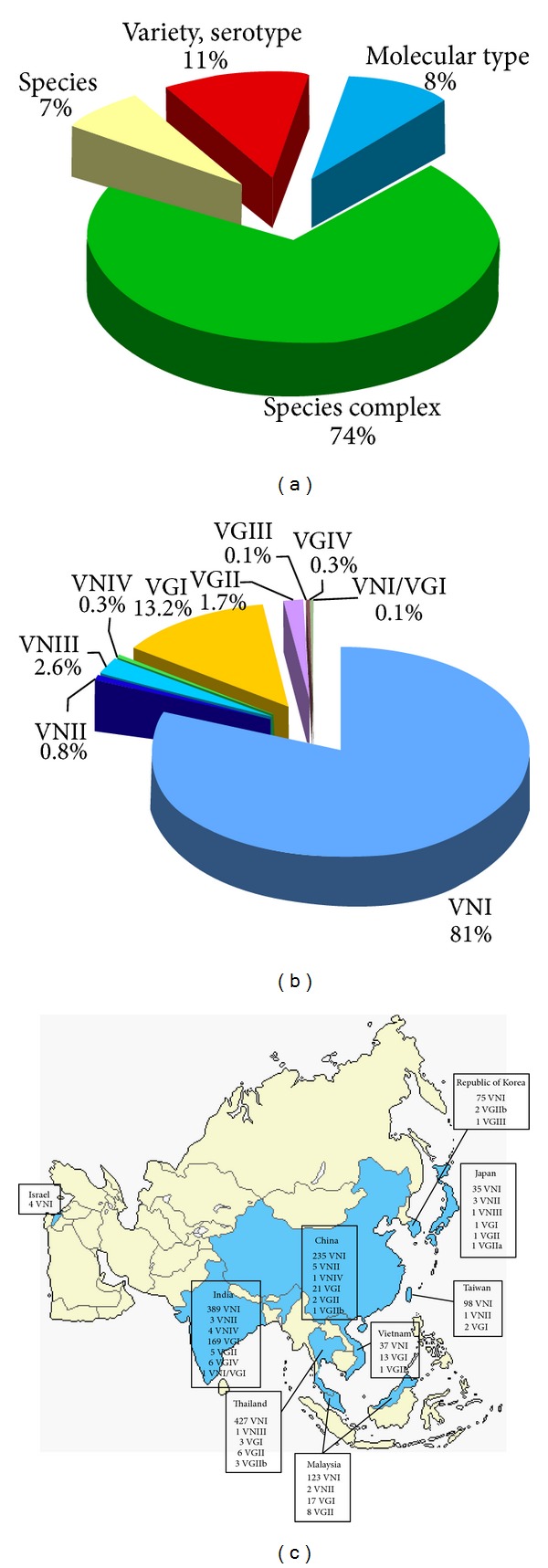
Percentage of *Cryptococcus neoformans* and *Cryptococcus gattii* isolates (*n* = 19,651) identified at species complex, species, variety/serotype, or molecular type level (a). Prevalence of the different VN and VG molecular types among the isolates identified at molecular type level (*n* = 1,708) (b). Geographic distribution of the molecular types identified in Asia (c). Molecular typing data have been combined from the following references: India [11, 14, 15, 28–36], China [8, 37–43], Thailand [11, 15, 44–48], Malaysia [49, 50], Vietnam [51], Taiwan [38, 52], Japan [38, 53, 54], Republic of Korea [55], and Israel [56].

The majority of the Asian isolates (74%) were identified just at species complex level, 7% at species level and 11% at variety/serotype level, while the molecular type was determined only in 8% (*n* = 1,708) (Figure 4). *C. neoformans* was the species prevalently isolated in Asia (*n* = 5,192), being *C. gattii* about tenfold less frequently (*n* = 682) isolated.

Eighty-one percent of the isolates belong to VNI and 13.2% to VGI molecular type. VGII molecular type is also represented, although in low percentages, in all the Asian countries included in the analysis, except for Israel and Taiwan. VNIII and VNIV molecular types are present in China and in India, as well as one VNIII isolate was reported from Thailand, whereas they are absent in the other Asian countries. Isolates belonging to VGIV molecular type and one interspecies VN1/VG1 hybrid were reported only in India [29, 34]. VGIII seems to be very rare or absent in Asia since only one isolate was detected in Republic of Korea [55] (Figure 4).

### 3.3. Africa

**Figure 5:**
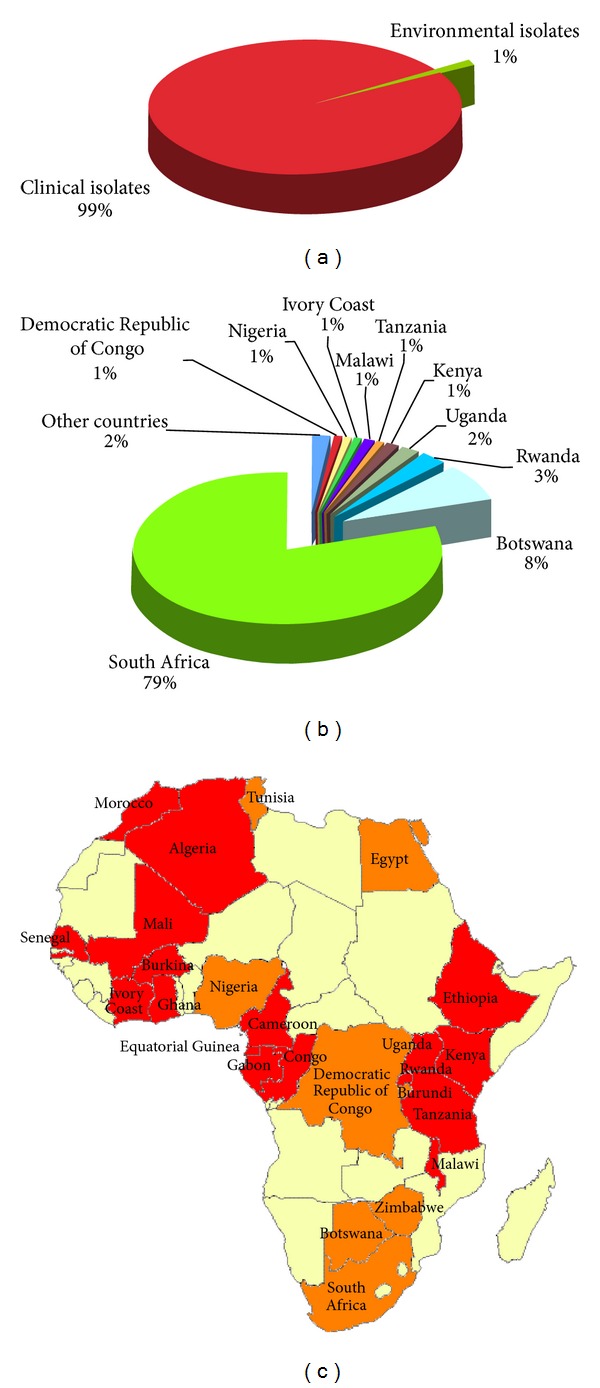
Percentage of *Cryptococcus neoformans* and *Cryptococcus gattii* isolates from clinical (*n* = 19,436) and environmental (*n* = 211) sources (a). Distribution of *Cryptococcus neoformans* and *Cryptococcus gattii* isolates in the different African countries (b). Map of the geographical distribution of the *Cryptococcus neoformans* and *Cryptococcus gattii* isolates in Africa (c). Clinical isolates were reported from red-colored countries, whereas both clinical and environmental isolates were reported from orange-colored countries.

**Figure 6:**
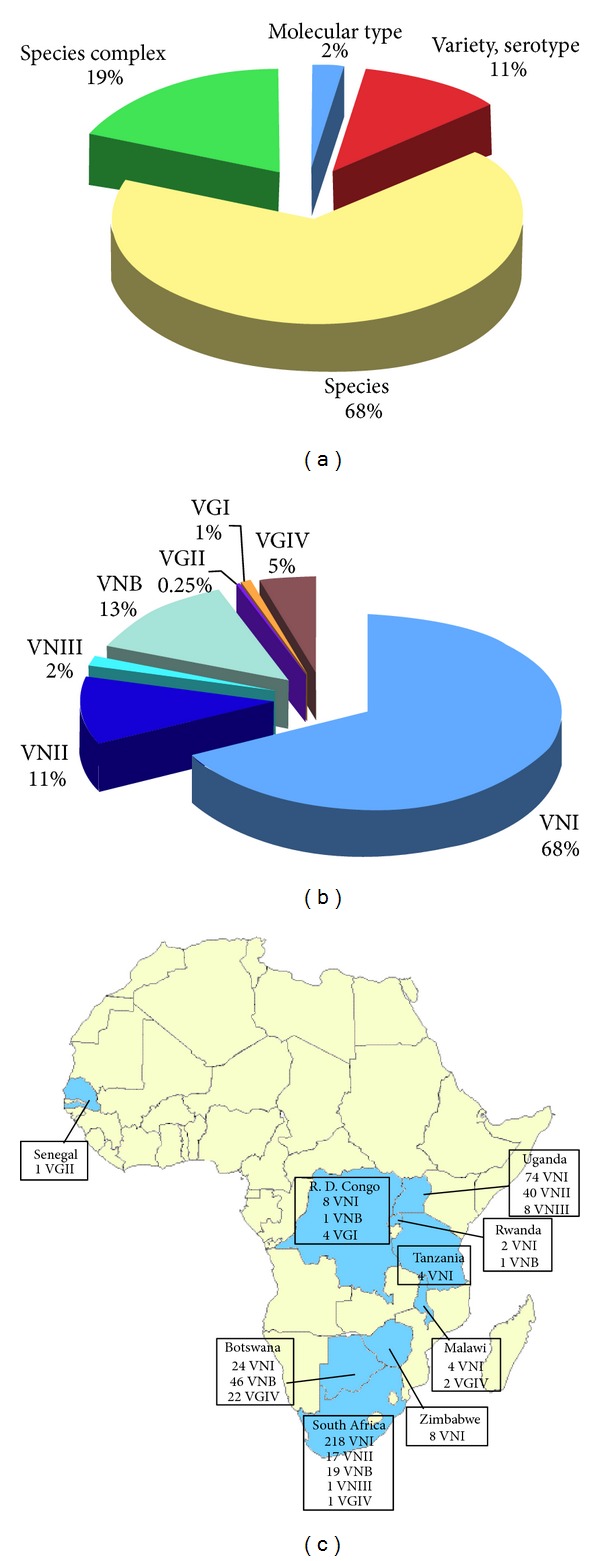
Percentage of *Cryptococcus neoformans* and *Cryptococcus gattii* isolates (*n* = 19,647) identified at species complex, species, variety/serotype, or molecular type level (a). Prevalence of the different VN and VG molecular types among the isolates identified at molecular type level (*n* = 505) (b). Geographic distribution of the molecular types identified in Africa (c). Molecular typing data have been combined from the following references: Senegal [14], Republic Democratic of Congo [11, 53], Uganda [53, 57], Rwanda [11], Tanzania [53], Malawi [53, 58, 59], Zimbabwe [11], Botswana [53, 58, 60, 61], and South Africa [11, 15, 38, 60, 62, 63].

Isolation of 19,753 *C. neoformans* and *C. gattii* strains was reported from 25 of the 58 African countries and mainly from South Africa (79%). Environmental surveys, carried out in eight countries (Tunisia, Egypt, Nigeria, Republic Democratic of Congo, Burundi, Zimbabwe, Botswana, and South Africa), recovered only 1% out the total reported isolates (Figure 5). *C. neoformans* was not only isolated from pigeon and other birds excreta but also from soil and house dust [60, 187, 188], as well as from trees such as *Eucalyptus camaldulensis*, mopane tree, and baobab [60, 189]. *C. gattii* was isolated from soil, *Eucalyptus camaldulensis*, and almond tree [60, 189]. Two veterinary isolates were also reported from two cases of cryptococcosis affecting South African cheetahs [38, 190]. The majority of the studies reported only the species of the isolates (68%), 19% were reported as *Cryptococcus* species complex, and 11% as variety or serotype (Figure 6). Molecular typing techniques were applied to identify 2% of the isolates (*n* = 505). Of these, 68% were molecular type VNI. VNII and VNIII represent 11% and 1% of the isolates, respectively, and have been reported only from Uganda and South Africa. Thirteen percent of the African isolates belongs to VNB molecular type, which was initially considered endemic of Botswana [53] but that is present also in South Africa, Rwanda, and Republic Democratic of Congo. In addition, a consistent population of the rare VGIV molecular type was isolated in Botswana and Malawi [58]. Only one isolate belonging to VGII and four belonging to VGI molecular type were reported from Senegal [14] and Republic Democratic of Congo [11], respectively, whereas VNIV was totally absent among the African isolates included in the present study (Figure 6).

### 3.4. Europe

**Figure 7:**
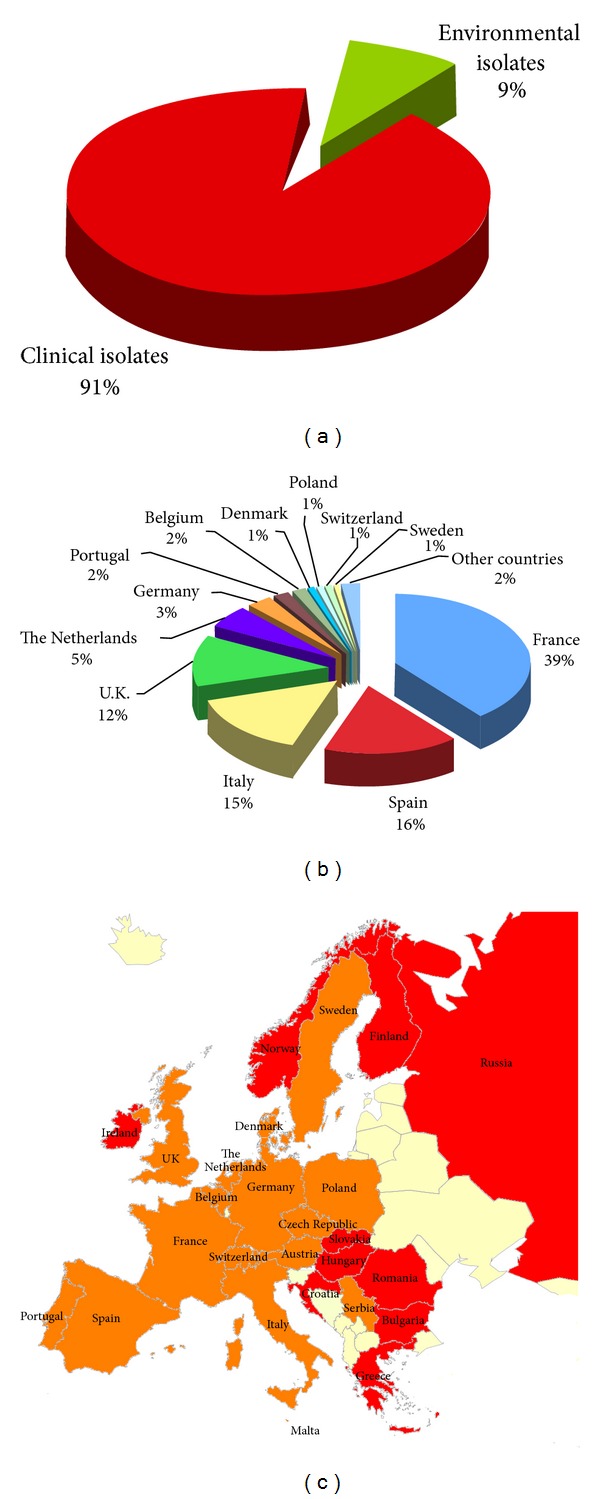
Percentage of *Cryptococcus neoformans* and *Cryptococcus gattii* isolates from clinical (*n* = 7,959) and environmental (*n* = 777) sources (a). Distribution of *Cryptococcus neoformans* and *Cryptococcus gattii* isolates in the different European countries (b). Map of the geographical distribution of the *Cryptococcus neoformans* and *Cryptococcus gattii* isolates in Europe (c). Clinical isolates were reported from red-colored countries, whereas both clinical and environmental isolates were reported from orange-colored countries.

The map in Figure 7 shows the European countries reporting the isolation of at least one *Cryptococcus* species complex strain. Data were lacking from some Balkan and Eastern European countries. The majority of the isolates were reported from France, Spain, Italy, and United Kingdom representing 82% out of the total (*n* = 8,736). Nine percent of the isolates were detected from environmental and veterinary sources (Figure 7). *C. neoformans* not only isolated from pigeon and other birds excreta, but also from bat guano and red fox faeces [65, 191, 192]. Veterinary isolates include strains recovered from cat, dog, magpie, and some isolates from striped grass mouse and degu living in a zoo [193–196]. Few *C. neoformans* strains were isolated from trees, namely, from *Eucalyptus camaldulensis* and oak tree [74, 197]. Most of the *C. gattii* natural isolates were from *Eucalyptus camaldulensis*, Douglas tree, carob tree, and stone pine [66, 74], whereas *C. gattii* animal infections were reported in a ferret and in some goats [65, 67].

**Figure 8:**
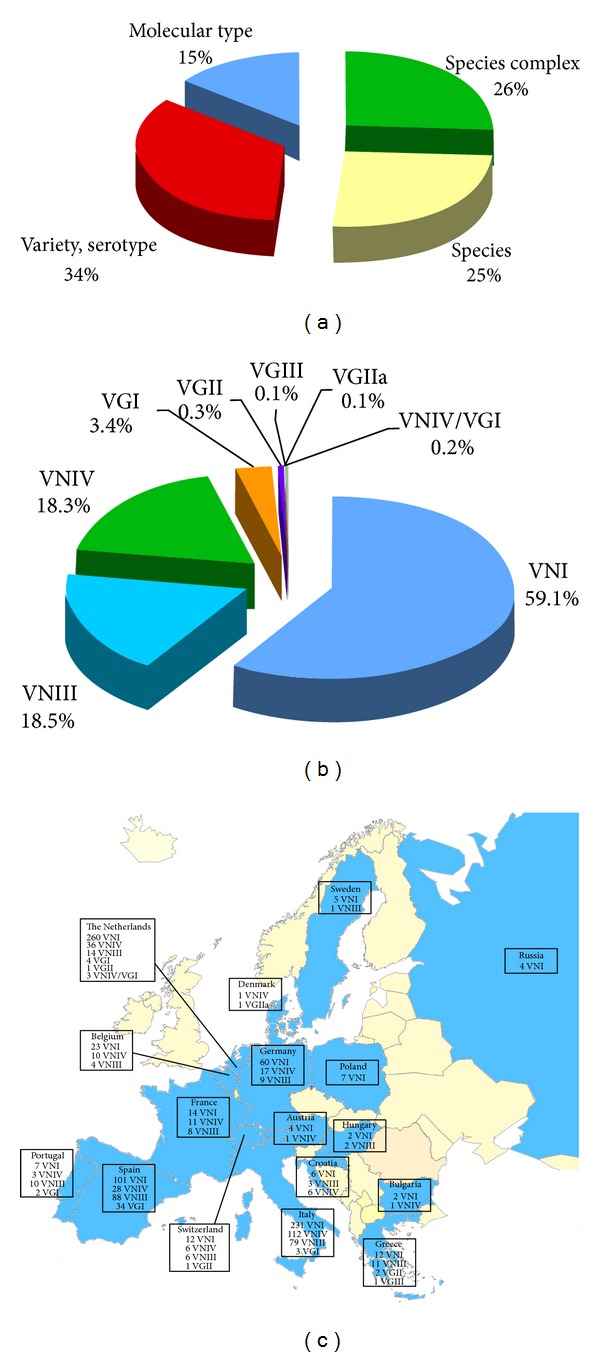
Percentage of *Cryptococcus neoformans* and *Cryptococcus gattii* isolates (*n* = 8,736) identified at species complex, species, variety/serotype, or molecular type level (a). Prevalence of the different VN and VG molecular types among the isolates identified at molecular type level (*n* = 1,269) (b). Geographic distribution of the molecular types identified in Europe (c). Molecular typing data have been combined from the following references: Portugal [14, 56], Spain [56, 64–71], France [11, 72], Belgium [11, 56], The Netherlands [11, 73–75], Switzerland [73, 76, 77], Austria [56, 77], Italy [38, 53, 56, 78–85], Germany [13, 77], Denmark [11, 86], Sweden [56], Bulgaria [56], Russia [56], Greece [34, 56, 87], Croatia [88], Hungary [56], and Poland [56].

Variety or serotype was determined in 34% of the European isolates, species in 25%, molecular type in 15%, while the 26% was reported as *Cryptococcus* species complex (Figure 8). A total of 6,371 isolates were identified as *C. neoformans* and 94 as *C. gattii*. 

European molecular typing data are shown in Figure 8. The majority of the isolates belong to VNI molecular type (59%), although VNIII and VNIV molecular types were also reported in most of the countries representing 18.5% and 18.3%, respectively*. C. gattii* molecular types distribution in Europe is not yet well defined. VGI is the prevalent molecular type, being 43 isolates reported from Portugal, Spain, Italy, and The Netherlands. Few VGII isolates were reported from Greece, Switzerland, The Netherlands, and Denmark, and only one VGIII isolate was found in Greece. A relevant observation is the absence of typing results from the United Kingdom, despite the fact that 12% of the European isolates were reported from this country (Figure 8).

### 3.5. Central and South America

**Figure 9:**
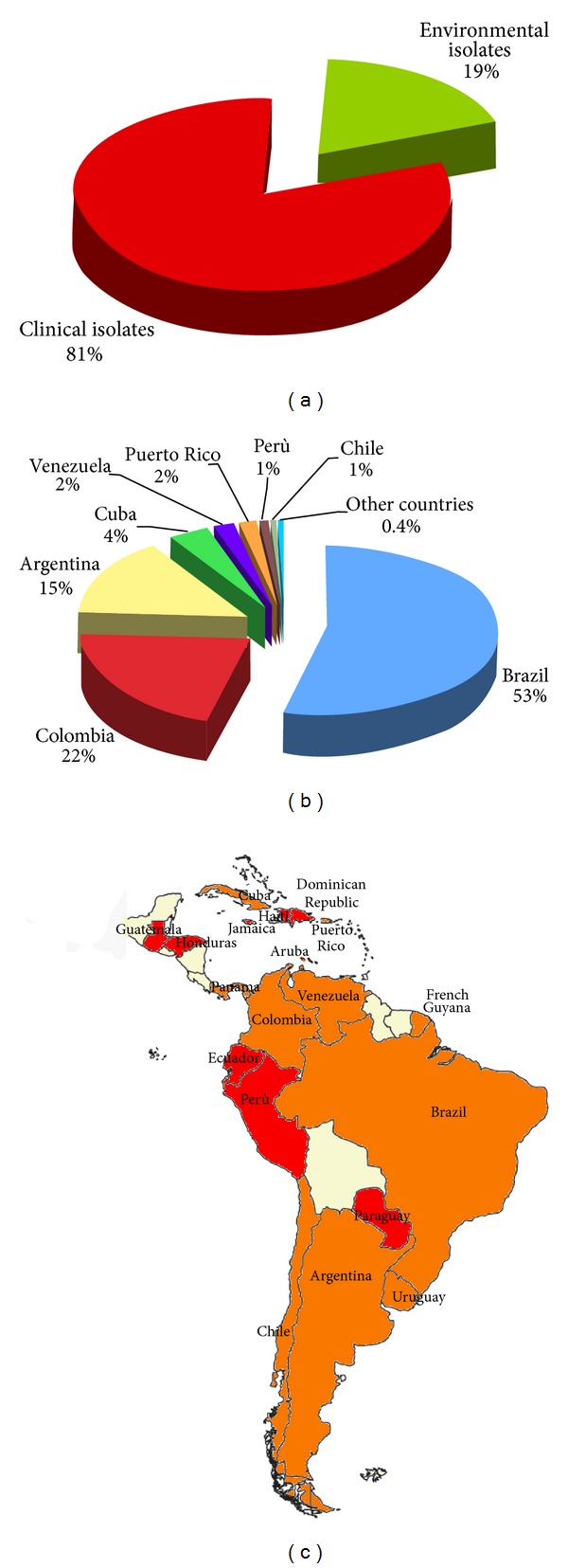
Percentage of *Cryptococcus neoformans* and *Cryptococcus gattii* isolates from clinical (*n* = 8,590) and environmental (*n* = 1,958) sources (a). Distribution of *Cryptococcus neoformans* and *Cryptococcus gattii* isolates in the different countries of Central and South America (b). Map of the geographical distribution of the *Cryptoccocus neoformans* and *Cryptococcus gattii* isolates in Central and South America (c). Clinical isolates were reported from red-coloredcountries, whereas both clinical and environmental isolates were reported from orange-colored countries.

Among the 10,548 *Cryptococcus* species complex isolates reported from Central and South America, the 53% were reported from Brazil, 22% from Colombia, 15% from Argentina, and a lower percentage from other countries. A total of 8,590 (81%) strains were isolated from clinical sources and 1,958 (19%) from environmental and veterinary sources (Figure 9). Natural *C. neoformans* isolates were detected from pigeon and other birds excreta, soil, dust, and contaminated dwellings [94, 98, 198–200], as well as from *Eucalyptus* tree, almond tree, kassod tree, pink shower tree, *Caesalpinia peltophoroides*, and *Anadenanthera peregrine* [90, 99, 102, 201, 202]. Some isolates were also recovered from insects, bull, and sheep [99, 203, 204]. *C. gattii* was isolated from soil, dust, and psittaciformes bird excreta [94, 103, 199], and from *Eucalyptus camaldulensis*, almond tree, kassod tree, pottery tree, jungle tree, *Corymbia ficifolia*, and *Cephalocereus royenii* [92, 95, 102, 205–208]. Animal infection due to *C. gattii* was reported in a cheetah, a goat, and some psittacine birds [11, 91, 209]. 

**Figure 10:**
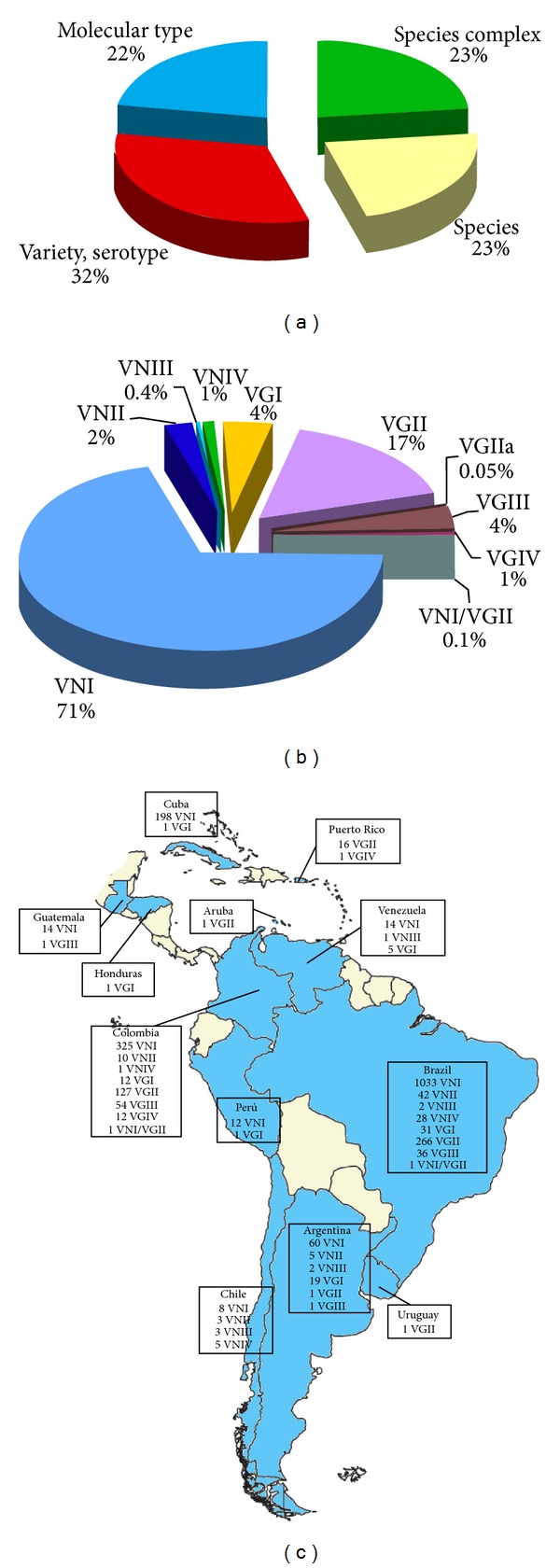
Percentage of *Cryptococcus neoformans* and *Cryptococcus gattii* isolates (*n* = 10,548) identified at species complex, species, variety/serotype, or molecular type level (a). Prevalence of the different VN and VG molecular types among the isolates identified at molecular type level (*n* = 2,345) (b). Geographic distribution of the molecular types identified in Central and South America (c). Molecular typing data have been combined from the following references: Guatemala [64], Honduras [11], Cuba [89–91], Puerto Rico [92], Aruba [11, 17], Venezuela [64], Colombia [29, 64, 93–96], Perù [64], Uruguay [11, 17], Brazil [11, 17, 29, 38, 46, 64, 97–118], Argentina [15, 64, 119], and Chile [64].

Seventy-seven percent of the isolates were identified at least at species level (32% as variety or serotype, 23% as species, and 22% as molecular type), and 23% were reported as *Cryptococcus* species complex (Figure 10). *C. neoformans* was recognized in 6,665 and *C. gattii* in 1,464 isolates. The combined analysis of the molecular typing data reported from Brazil (1,439 isolates) showed that all the molecular types, except for VGIV, are represented in this country. The majority of the isolates in Brazil belong to VNI (*n* = 1033) molecular type followed by VGII (*n* = 266), while VNII, VNIV, VGI, and VGIII occurred in a lower but similar percentage. Two isolates of VNIII as well as one VNI/VGII hybrid were also reported. In Colombia (542 isolates), the prevalence of molecular types was similar to that observed in Brazil, except for VGIII, which occurred in a higher percentage than VNII, VGI, and VGIV, as well as VNIV, that was recognized only in one isolate. VNIII AD hybrids seem to be absent in Colombia. Data from Argentina (94 isolates) showed that the VGI molecular type is the prevalent genotype among *C. gattii* isolates, in contrast to that observed in Brazil and Colombia, where it is the VGII. In Cuba, 198 VNI isolates were detected, whereas only one isolate was VGI. On the contrary, in the near Puerto Rico, only *C. gattii* isolates (16 VGII and one VGIV) were reported. Finally, all the four *C. neoformans* molecular types were reported from Chile, but no *C. gattii* isolates were found (Figure 10).

### 3.6. North America

**Figure 11:**
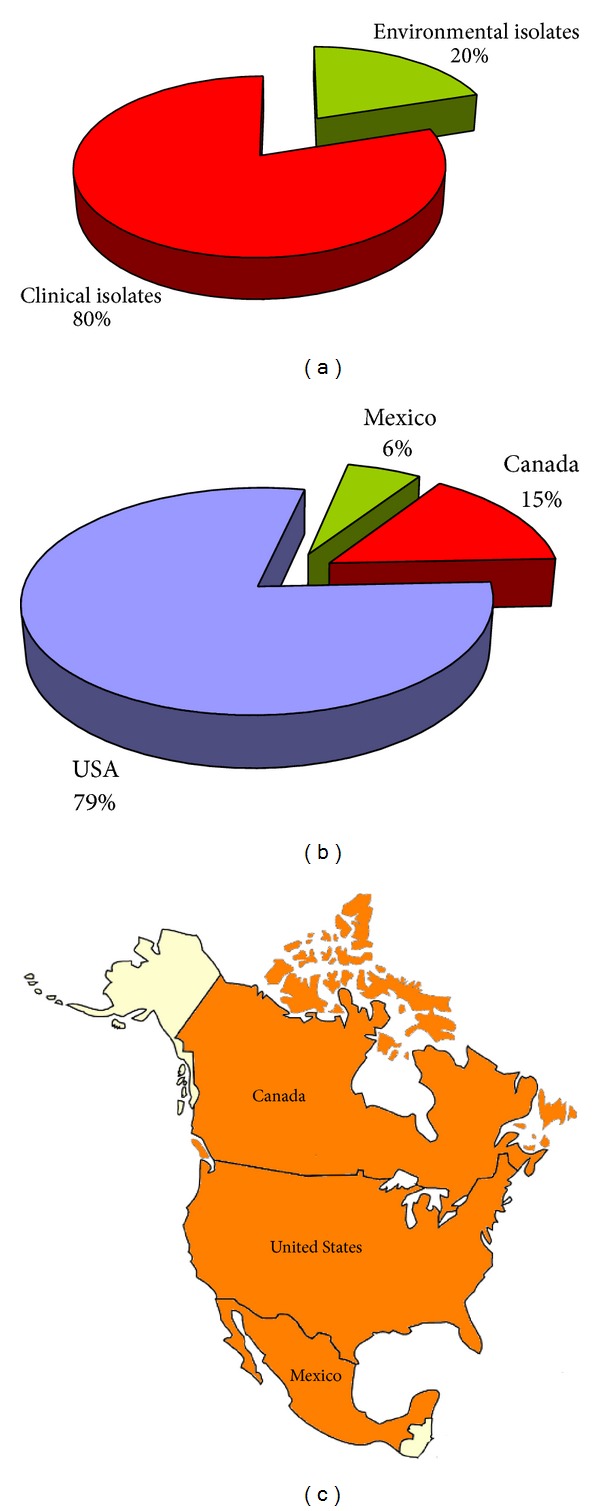
Percentage of *Cryptococcus neoformans* and *Cryptococcus gattii* isolates from clinical (*n* = 6,248) and environmental (*n* = 1,674) sources (a). Distribution of *Cryptococcus neoformans* and *Cryptococcus gattii* isolates in the different countries of North America (b). Map of the geographical distribution of the *Cryptococcus neoformans* and *Cryptococcus gattii* isolates in North America (c). Clinical isolates were reported from red-colored countries, whereas both clinical and environmental isolates were reported from orange-colored countries.

A total of 7,922 *C. neoformans* and *C. gattii* isolates were reported from the USA (79%), Canada (15%), and Mexico (6%). Eighty percent of the isolates were from clinical sources, whereas 20% were recovered from the environment and animals (Figure 11). Pigeon droppings were the main source for *C. neoformans* isolation [210, 211], although, in Mexico, it was also isolated from fruit and vegetables [212]. Three *C. neoformans* isolates (two in Canada and one in the United States) caused infection in ferrets [170, 213]. Isolation of *C. gattii* from the environment and from animals was widely reported from Canada during the monitoring of the Vancouver Island *C. gattii* outbreak. Soil, trees, and animals living in Vancouver Island (dogs, cats, horses, ferrets, and birds) resulted colonized or infected with this pathogen [121]. Outside Canada, VGIIa isolates were found in the environment and animals (air, water, soil, tree, cats, dogs, alpacas, and parrots) in Oregon and Washington State [123, 124], and one VGI strain was isolated from *Eucalyptus camaldulensis* in Mexico [214].

**Figure 12:**
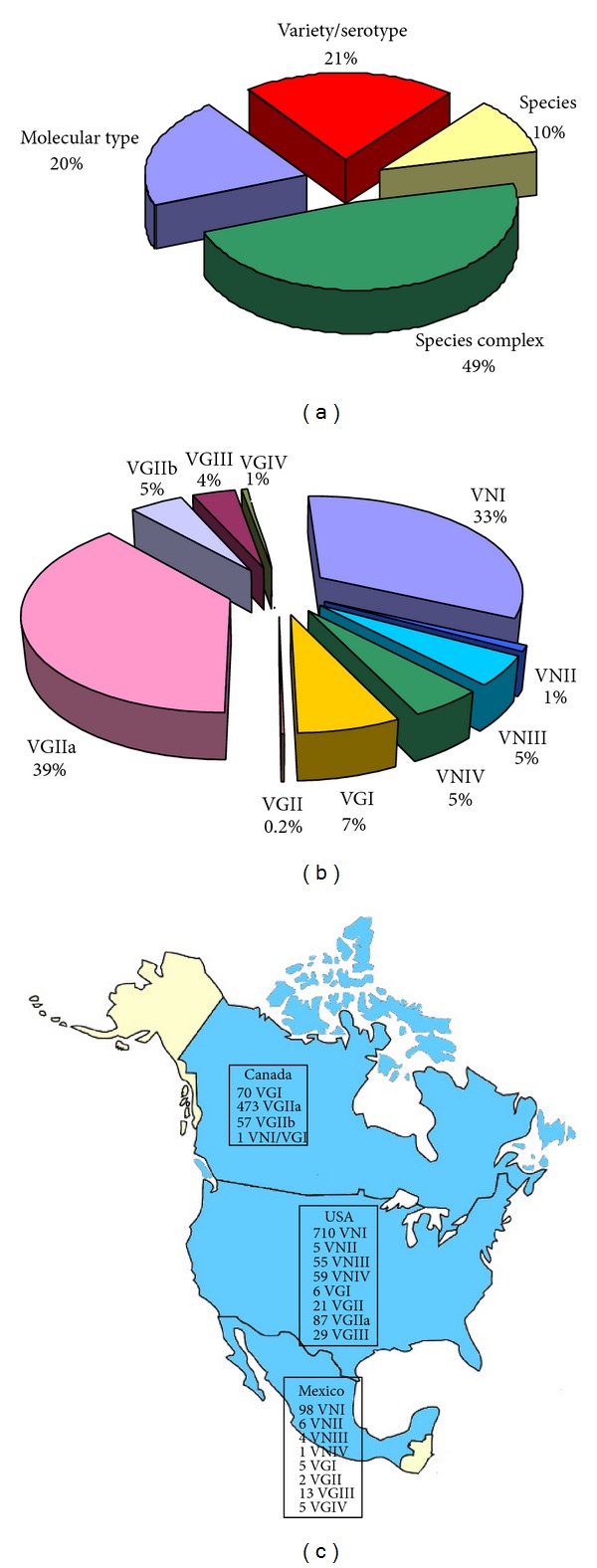
Percentage of *Cryptococcus neoformans* and *Cryptococcus gattii* isolates (*n* = 7,922) identified at species complex, species, variety/serotype, or molecular type level (a). Prevalence of the different VN and VG molecular types among the isolates identified at molecular type level (*n* = 1,707) (b). Geographic distribution of the molecular types identified in North America (c). Molecular typing data have been combined from the following references: Mexico [64, 120], Canada [121, 122], of the United States [53, 58, 123–130].

Almost half of the isolates (49%) reported from North America were identified only as *Cryptococcus* species complex, 10% were identified at species level, while variety or serotype was reported for 21%. Molecular type was determined in 20% of the isolates (*n* = 1,707) (Figure 12). Despite the fact that *C. neoformans* was more frequently isolated in North America than *C. gattii* (3,148 *versus* 885 isolates, resp.), 39% of the isolates identified by molecular techniques belong to VGIIa molecular type. This is due to the extensive effort produced to discover the cause of Vancouver Island outbreak which, at present, includes 473 VGIIa, 57 VGIIb, and 70 VGI isolates [121]. In addition, a recent study has reported the infection of a Canadian patient with an interspecies VNI/VGI AB hybrid strain [122]. VNI was the prevalent molecular type in both the United States and Mexico, where VNII, VNIII, VNIV, and VGI are also present in lower percentages. VGII and VGIIa *C. gattii* molecular types were reported from the Northwest Pacific Coast of United States, while VGIII was reported more frequently from Mexico and Southern California [64, 120, 125]. Five VGIV isolates were reported from Mexico [64, 120], although this molecular type is absent in Canada and in the United States.

## 4. Concluding Remarks

The present combined analysis shows that about 68,811 *C. neoformans*/*C. gattii* isolates were reported in the world till now. The majority of the isolates were reported from Asia and Africa (19,651 and 19,647 isolates, resp.), followed by Central and South America (*n* = 10,548), Europe (*n* = 8,736), North America (*n* = 7,922), and Oceania (*n* = 2,518). The countries where the isolates were prevalently isolated are South Africa (*n* = 15,361), China (*n* = 9,736), USA (*n* = 6,198), and Brazil (*n* = 5,709). On the contrary, data are completely lacking from many countries of Africa, Asia, and Eastern Europe. United States is the country where the environment was more extensively surveyed (1089 isolates), followed by Brazil (*n* = 893), Australia (*n* = 758), Colombia (*n* = 742), and India (*n* = 569). Although 723 environmental isolates were also reported from Canada, they are not representative of the whole country since they were recovered during the monitoring of a restricted territory such as Vancouver Island area. In 38.5% of the isolates (*n* = 26,473) reported in the literature, the species was not determined, whereas among the isolates identified at least at species level (61.5%, *n* = 42,338), *C. neoformans* was about eightfold more frequently isolated than *C. gattii* (88.6% *versus* 11.4%). The *C. neoformans*/*C. gattii* ratio is variable for each continent being 68 : 1 in Europe, 33 : 1 in Africa, 7.6 : 1 in Asia, 4.5 : 1 in Central and South America, 3.5 : 1 in North America, and 1 : 1.5 in Oceania, where *C. gattii* is the prevalent species isolated.

Molecular type was determined for 8,077 isolates (12%) representing only a part of the world countries. Molecular data are absent from large parts of Africa, Asia, Eastern Europe, as well as from United Kingdom, Ireland, Norway, and Finland. VNI is the prevalent molecular type worldwide except in Australia and Papua New Guinea, where it is VGI. This latter molecular type was also found in 13.2% of the Asian, in 7% of the North American, in 4% of the Central and South American, and in 3.4% of the European isolates, while only four VGI strains have been reported from Africa. VNII is a rare molecular type which is reported from all the continents, except from Europe, in low percentages. However, a recent MLST study carried out in Italy has showed the presence of one VNB and three VNII strains also among a group of Italian clinical isolates, suggesting that these populations are underestimated in the European continent [215]. In addition, two VNB isolates from Brazil and Colombia, previously reported as VNII, were recognized by MLST analysis, confirming that VNB molecular type is not endemic of Southern Africa [22]. The distribution and prevalence of the VGII molecular type is relevant to elucidate the origin of the Vancouver Island and Northwest Pacific Coast *C. gattii* outbreak. The present analysis has identified four main reservoirs of VGII molecular type: Brazil (266 isolates), Colombia (*n* = 127), Australia (*n* = 121), and Puerto Rico (*n* = 16). These data confirm the hypotheses suggested by other authors that the Vancouver outbreak could be originated from Australia [14] or from South America [22]. The VGIII molecular type has been prevalently detected in Latin American countries, including Mexico and Sothern California (134 isolates). In the other continents, VGIII is very rare, counting one isolate in Republic of Korea, one in Greece, and six in Oceania. The abundance of VNIII AD hybrids seems to be strictly related to the presence of VNIV molecular type. In Europe and in the USA, where the frequency of isolation of VNIV strains is higher than in other geographical areas (18% and 6%, resp.), a similar percentage of VNIII isolates has been observed, suggesting that in these regions hybridization between VNI and VNIV populations is occurring. VGIV molecular type was reported from Southern Africa (*n* = 24), India (*n* = 6), Colombia (*n* = 12), and Mexico (*n* = 5), but a recent MLST study, comparing these isolates, has revealed that Indian and Southern African isolates are strictly correlated and different from those from South America [34]. Finally, the interspecies *C. neoformans*/*C. gattii* hybrids have been rarely reported from different geographical areas, namely, one from India, one from Colombia, one from Brazil, one from Canada, and three from The Netherlands. However, due to the difficulty to identify these hybrids, it is likely that their prevalence is underestimated. 

In conclusion, the present study describes the state of the art of *C. neoformans* and *C. gattii* genotyping by a detailed representation of the geographical distribution of the major molecular types, which could be a useful tool to start new epidemiological surveys on the basis of the present knowledge. 

## Figures and Tables

**Table 1:** Genotype nomenclature adopted by the main molecular typing techniques and correspondence to standard nomenclature.

Species/variety	Standard nomenclature	PCR-fingerprinting M13 [15]	PCR-fingerprinting (GACA)_4_[155]	AFLP [11]	MLST [14, 53]	Reference strains
*C. neoformans* var. *grubii *	VNI	VNI	VN6	AFLP1	VNI	ATCC MYA-4564 [15]
	VNII	VNII	VN6	AFLP1A or AFLP1B	VNII	ATCC MYA-4565 [15]
	VNB	VNI or VNII	VN6	AFLP1A or AFLP1B	VNB	bt1, bt131, bt100 [53]
*C. neoformans* var. *neoformans *	VNIV	VNIV	VN1	AFLP2	—	ATCC MYA-4567 [15]
Inter-varietal AD hybrids	VNIII	VNIII	VN3 or VN4	AFLP3	—	ATCC 32045 [168]
*C. gattii *	VGI	VGI	—	AFLP4	VGI	ATCC MYA-4560 [64]
	VGII	VGII	—	AFLP6	VGII	ATCC MYA-4561 [64]
	VGIII	VGIII	—	AFLP5	VGIII	ATCC MYA-4562 [64]
	VGIV	VGIV	—	AFLP7	VGIV	ATCC MYA-4563 [64]
Inter-species hybrids	VNI/VGI	VNI/VGI	—	AFLP9	—	CBS 10496 [122]
	VNIV/VGI	VNIV/VGI	—	AFLP8	—	CBS 10488 [122]
	VNI/VGII	VNI/VGII	—	—	—	WM 05-272 [29]

ATCC: American Type Culture Collection (http://www.atcc.org/); CBS: Centraalbureau voor Schimmelcultures (http://www.cbs.knaw.nl/); WM: Westmead Millennium Institute, Sydney, Australia; bt: isolate from Botswana.
